# Digital disease detection and participatory surveillance: overview and perspectives for Brazil

**DOI:** 10.1590/S1518-8787.2016050006201

**Published:** 2016-04-27

**Authors:** Onicio B Leal-Neto, George S Dimech, Marlo Libel, Wanderson Oliveira, Juliana Perazzo Ferreira

**Affiliations:** I Epitrack eHealth. Recife, PE, Brasil; II Programa de Pós-graduação em Saúde Pública. Centro de Pesquisas Aggeu Magalhães. Fundação Oswaldo Cruz. Recife, PE, Brasil; III Diretoria Geral de Doenças e Controle de Agravos. Secretaria de Saúde do Estado de Pernambuco. Recife, PE, Brasil; IV Skoll Global Threats Fund. San Francisco, CA, USA; V Coordenação Geral de Vigilância e Resposta a Emergências em Saúde Pública. Secretaria de Vigilância da Saúde. Ministério da Saúde. Brasília, DF, Brasil; VI Programa de Pós-graduação em Ciência da Computação. Universidade Federal de Pernambuco. Recife, PE, Brasil

**Keywords:** Public Health Surveillance, Mobile Applications, trends, Information Technology, Science, Technology and Society

## Abstract

This study aimed to describe the digital disease detection and participatory surveillance in different countries. The systems or platforms consolidated in the scientific field were analyzed by describing the strategy, type of data source, main objectives, and manner of interaction with users. Eleven systems or platforms, developed from 1996 to 2016, were analyzed. There was a higher frequency of data mining on the web and active crowdsourcing as well as a trend in the use of mobile applications. It is important to provoke debate in the academia and health services for the evolution of methods and insights into participatory surveillance in the digital age.

## RESUMO

O objetivo deste estudo foi descrever a detecção digital de doenças e vigilância participativa em diferentes países. Os sistemas ou plataformas consolidados no meio científico foram analisados por descrição da estratégia, do tipo de fonte de dados, dos objetivos principais e da forma de interação com os usuários. Foram analisados 11 sistemas ou plataformas, desenvolvidos entre 1996 e 2016. Observou-se maior frequência de mineração de dados na *web* e *crowdsourcing* ativo e tendência no uso de aplicativos móveis. É importante provocar o debate nos âmbitos acadêmico e dos serviços de saúde para evolução dos métodos e percepções sobre a vigilância participativa na era digital.

## INTRODUCTION

Epidemiological studies applied to health surveillance in the last two decades have begun a recruitment of new methodologies to investigate outbreaks or track trends in infectious diseases, aiming at the early identification of outbreaks and infectious diseases^1^. Currently, many digital platforms, such as HealthMap, Google Flu Trends, and Flu Near You, allow visualizing epidemiological scenarios around the world, providing data on diseases for the population, travellers and health services^7^. When the International Health Regulations (IHR) were implemented, they already counted on the evolution of transport systems that help individuals travel all over the planet. These systems make infectious diseases spread quicker, demanding greater agility in identifying these risks^2^. In addition, one of the points updated in the last version of the IHR was the use of unofficial sources (for example, the general media) to detect rumors about possible outbreaks or cases of diseases considered public health emergencies^2^. Using these unofficial sources comes down largely to information available on the internet, whether produced and distributed by news websites and other websites, or collectively by users in social media – a movement known as crowdsourcing^3^. The publication and dissemination of content produced by users and related to the epidemiology of diseases have been characterized by Participatory Epidemiology^6^ and its study may be defined as Infodemiology and Infoveillance, respectively, Information Epidemiology and Information Surveillance^4^. Computational techniques have enabled data mining in cyberspace, in other words, analyzing semantics and keywords scattered on the internet, linked to texts of epidemiological relevance, capturing and counting sets of words, indicating trends^4^. Research in more specific niches, unaffected by the methods described previously due to technical issues, is also cited as an important source for public health, coinciding with the epidemic curves of the health harms. One example is mining in online social networks as Twitter^4^ ^,^ ^6^.

Despite being recognized as a center of computer development in health, Brazil presents shy results in scientific production related to digital detection of diseases. The Country has successful experiences using data mining in social networks and participatory surveillance related to studying the epidemiology of dengue, as the projects *Observatório da Dengue* (Dengue Observatory) and *Dengue na Web* (Dengue in the Web)^4^ ^,^ ^7^. However, other diseases of acute clinical features and requiring a rapid detection were not covered by strategies of this type, indicating the lack of studies geared to notifiable diseases. Considering this and the Brazilian tourism, commercial, and industrial development, strategies like these had priority in implementation. Large events such as the FIFA World Cup demand optimized advance preparation of the healthcare sector to mitigate potential infectious risks, and it resulted in the project *Saúde na Copa* (Healthy Cup)^7^. Since this successful experience took place, Brazil has remained investing efforts and collaborations to continue the participatory surveillance, culminating in the launch of the platform *Guardiões da Saúde* (Guardians of Health), in 2016.

The objective of this study was to describe the digital disease detection and participatory surveillance in different countries from 1996 to 2016.

## METHODS

From October 2013 to March 2016, a descriptive analysis was performed using categories based on the objects of study, divided into: (1) Digital disease detection on the web; (2) Participatory surveillance (active crowdsourcing); and (3) Twitter (passive crowdsourcing). The first category describes experiments using active search for epidemiologically relevant data on the internet. The second one, experiments in which individuals are the primary source of information for building epidemiological scenarios. The final one presents the research related to collecting data of epidemiological relevance on Twitter. To specify the object of study, only experiments of consolidated recognition in the scientific world, measured by citations in articles on this theme, were discussed.

To characterize the analysis of the platforms, we described the strategy, the data source type (primary for data collected directly from users and secondary for data collected in the cyberspace), and the main goals of each system and platform of interaction with users (website for collection, record, and search for information on websites and mobile application for collection, record, and search for information using mobile devices as tablets and smartphones).

## RESULTS

Table describes the strategy of 11 systems or platforms of digital detection of diseases. Regarding the characterization of systems, we observed a predominance of North American countries, higher frequency of epidemiologically relevant data mining on the web and active crowdsourcing, use of primary and secondary data sources, and a trend in the use of mobile applications to collect, record, and search information. All platforms require users to register so they can send and have access to information, but are free of cost.

**Table:** Description of the systems or platforms of digital detection of diseases.

Title	Country, base year	Strategy type	Data source	Main objectives	Interaction platform
ProMED	USA, 1996	A	Secondary	Collecting data in cyberspace related to diseases and conditions.	Website and mobile application
GPHIN	Canada, 1997	A	Secondary	Collecting data in cyberspace related to diseases and conditions.	Website
InfluenzaNet	The Netherlands and Belgium, 2003 Portugal, 2005 Italy, 2008 UK, 2009	B	Primary	Collecting information on influenza-like illness data, made available to the population.	Website and mobile application
HealthMap	USA, 2006	A, B	Primary and secondary	Spatializing epidemiologically relevant information, made available to the population via web.	Website and mobile application*
MedISys	Italy, 2007	A	Secondary	Collecting data in cyberspace related to diseases and conditions.	Website
Salud Boricua	USA (for Puerto Rico only), 2008	B	Primary	Spatializing information on acute febrile syndrome (dengue fever, influenza, leptospirosis) data, made available to the population.	Website
Flu Near You	USA, 2011	B	Primary	Spatializing information on influenza-like illness data, made available to the population.	Website and mobile application
*Dengue na Web* (Dengue in the Web)	Brazil, 2011	B	Primary	Spatializing information on data related to dengue fever.	Website
*Observatório da Dengue* (Dengue Observatory)	Brazil, 2011	C	Primary	Spatializing tweets related to dengue fever.	Website
*Saúde na Copa* (Healthy Cup)	Brazil, 2014	A, B	Primary and secondary	Detecting possible changes in the epidemiological pattern of acute disease occurrence in 12 Brazilian host cities during the 2014 FIFA World Cup.	Website and mobile application
*Guardiões da Saúde* (Guardians of Health)	Brazil, 2016	B	Primary and secondary	Detecting in advance aggregates of cases of diarrhoeal, respiratory, and exanthematic syndromes in Brazil.	Website and mobile application

A: Mining of epidemiologically relevant data on the web; B: Participatory surveillance (Active crowdsourcing); C: Data mining on Twitter (Passive crowdsourcing)

* Made by the application Outbreaks Near Me.

Seven of those platforms showed a potential use in Brazil, where some of them are already part of the routines of the *Centros de Informações Estratégicas de Vigilância em Saúde* (CIEVS – Centers for Strategic Health Surveillance Information) of health departments of capitals and states and the Ministry of Health.

## DISCUSSION

With the current epidemiological surveillance information flow, sick individuals are known by the surveillance only when they access a service where, after receiving the suspected diagnosis, they can be notified as possible cases. However, the time between illness and notification can impact public health, e.g., if the case is exposed to several people susceptible to that disease^3^ ^-^ ^7^. On the other hand, this gap can be filled both by investigating unofficial sources such as social networks, where users often post their routine situations (passive participation), or using specific instruments for syndrome data collection (active participation). In both cases, the time gap previously mentioned is overcome by obtaining relevant information^3^ ^-^ ^7^.

Regarding data mining in social networks, of passive participation^5^ ^,^ ^6^, an issue to be considered is: what is the extent of the freedom of epidemic intelligence services in the search for information in these environments, since users are unaware that what they post is considered suspect?

Obviously the constitution did not provide for such evolution of the technologies, but attempts to adapt came with the movement for the *Marco Civil da Internet* (Brazilian Civil Rights Framework for the Internet), which guides users’ principles, guarantees, rights and duties^6^ ^,^ ^7^. In a scenario where the early detection of suspected cases and rumors is important, should these issues be above the minimization of the risk of spreading any infectious disease? Research ethics committees need to empower themselves on the themes, know the various instruments and see the horizon of possibilities in the field, for the good judgement of the studies that will produce the future instruments of this area.

Another important aspect is the current limitations to validate information, with aspects of cultural changes in the collaborative thinking of society to build sources of information with popular participation and social control. In fact, when detecting threats, suspicions or rumors for these types of information sources, risks can only be mitigated with local teams of epidemiological research. However, investment in these task forces may be questionable if strategies like this are not recognized as complementary to traditional sources, as part of the routine epidemiological surveillance flow.

There are several points to be discussed and the role of this communication is not to exhaust the subject, but to instigate debate in academic and health services so they can know and approach new methods and opportunities created by participatory surveillance in the digital age. This approach is still new in Brazil, but there are already dozens of successful experiences abroad, indicating a trend that will strengthen in the coming years.
